# Exploring the association between adolescent psychotic-like experiences and components of social performance using a multi-level virtual reality paradigm

**DOI:** 10.1007/s00127-025-02871-x

**Published:** 2025-03-17

**Authors:** Grace Kiernan, Pauline Kohl, Ekincan Tas, Frederic Berg, Mario Wolf, Phuong-Mi Nguyen, Lucia Valmaggia, Mar Rus-Calafell

**Affiliations:** 1 Mental Health Research and Treatment Centre, Faculty of Psychology, Ruhr University Bochum, Bochum, Germany; 2 Department of Digital Engineering, Ruhr Uaniversity Bochum, Bochum, Germany; 3 Orygen, Centre for Youth Mental Health, The University of Melbourne, Melbourne, Australia; 4 Department of Psychiatry, KU Leuven, Leuven, Belgium; 5 Department of Psychology, Institute of Psychiatry, Psychology & Neuroscience, King’s College London, London, SE5 8AF UK; 6 German Center of Mental Health (DZPG), Partner Site Bochum/Marburg, Bochum, Germany

**Keywords:** Psychosis risk, Psychotic-like experiences, Social performance, Virtual reality

## Abstract

**Background:**

Despite evidence linking psychotic-like experiences (PLEs) and social functioning deficits in youth at the risk of transitioning to psychosis, this association remains poorly understood. To address this, we explored the association between components of social performance and PLEs in adolescents aged 13–18 using a novel virtual reality (VR) paradigm for real-time assessment.

**Methods:**

Adolescents (*N* = 146) aged 13–18 were recruited as part of a larger cohort study conducted by the same research group (YVORI_PRO) and invited to participate via the following criteria: those reporting highly indicative positive PLEs (HIP, *N* = 88) and those reporting no or less indicative PLEs (no-HIP, *N* = 58). Self-report, behavioural and physiological components of social performance were collected using a portable VR headset and a medical wristband. Participants entered a virtual recreational area with three levels of social ambiguity and were encouraged to interact with avatars. MANOVA was performed to check for overall group differences and repeated measures ANOVAs were conducted to examine the effects of group and level of ambiguity, as well as their interaction, on daily social performance.

**Results:**

During virtual social interactions, adolescents with HIP reported higher levels of anxiety, fear of negative evaluation (FNE) and avoidance than the no-HIP group. No significant difference between groups was found for self-confidence. With increasing social ambiguity in VR, anxiety, FNE and avoidance increased in both groups, while self-confidence decreased. No significant group differences were found in behavioural or physiological components of social performance. Interpersonal distance and pulse rate increased significantly with increasing level of ambiguity, but pulse rate variability and skin conductance did not.

**Conclusion:**

The results suggest that adolescents with HIP may present specific difficulties related to social performance, which may carry additional psychosis risk. The new VR social scenario appears to be an acceptable, safe and effective tool to measure social performance in adolescents experiencing PLEs.

## Introduction

Research substantiates the concept that psychosis exists on a continuum, with people in the general population experiencing phenomena that resemble overt psychotic symptoms [1, 2]. While psychotic-like experiences (PLEs) are particularly prevalent in adolescence [3] and mostly transient [4], they can indicate a heightened risk for developing not only psychosis, but also other mental health disorders [3, 5, 6]. Despite advances in understanding PLEs, heterogeneity in the type and combination of experiences poses a challenge [7], and there remains a lack of consensus regarding phenomenology and assessment [8]. A systematic review on definitions of PLEs [9] shows that PLEs has been used as an umbrella term for phenomena such as out-of-the-body experience, magical ideation, cognitive disorganization and introvertive anhedonia. However, recent definitions of PLEs have typically encompassed subclinical levels of hallucinations and delusions while neglecting other types of PLEs [3, 10, 11].

Focusing on hallucination-like experiences and delusions is supported by findings showing that self-report measures of these two experiences reliably predict interviewer-rated PLEs in child and adolescent samples, while other types of PLEs do so to a much lesser extent [12, 13]. Furthermore, among 23 identified PLEs, hallucination-like experiences, delusions and catatonic-like symptoms have been found to be the most predictive of developing a psychotic disorder [14]. These findings raise the question whether adolescents experiencing subclinical hallucinations and delusions may constitute a distinct subgroup on the psychosis continuum. Further research is needed to examine potential subtypes of PLEs and their psychopathological significance [15].

Functioning is understood to be the ability to perform roles in different life domains [16]. Impairment in functioning is recognized as one of the most robust predictors of transition to psychosis in individuals at clinical high-risk [17]. In adolescents, the experience of PLEs has been associated with poorer functioning [18, 19], raising the question whether this population may be at a heightened risk of transitioning to psychosis. However, research into specific areas of dysfunction among adolescents experiencing PLEs is still in its infancy [20, 21].

One key area of dysfunction is social impairment, which can be assessed through indicators of social performance, such as social avoidance, anxiety [22], and fear of negative evaluation [23]. These factors are critical in determining an individual’s capacity to initiate and engage in social interactions. Notably, these components are often negatively impacted in individuals experiencing PLEs [24–27]. More negative social comparisons, also described as lower social rank, have been shown to increase paranoid ideation [28, 29], raising the question whether deterioration in social performance may cause or intensify PLEs and vice versa, increasing risk for transitioning to psychosis. While in those at clinical high-risk (CHR), this relationship is established [17], it remains unclear how and when deterioration in social performance appears in the context of PLEs in adolescents and the resulting risk.

Virtual reality (VR) enables observation of real-time interactions and manipulation of the environment in a controlled and safe setting [30, 31]. Components of social performance such as those already mentioned can be reliably assessed in VR [32, 33], though there are some variables that are not consistently assessed in prior VR studies, such as level of immersion. Manipulating social stressors in VR such as population or ethnic density or hostility [34] can influence components of social performance such as anxiety [35], fear of negative evaluation [36] and social avoidance [37]. VR environments also allow for reliable measurements of behavioural parameters of social performance. such as interpersonal distance (IPD) [38]. Interestingly, greater IPD-keeping has been shown to indicate greater social impairment [39]. However, this measure has only been explored in adult populations with psychosis and CHR individuals [40], where those with psychosis kept greater IPD. It remains unknown though whether adolescents experiencing PLEs also show similar results. Increases in anxiety have been related to increased avoidance behaviour [41], raising the question whether increases in social ambiguity might also affect IPD in adolescents.

In addition to behavioural parameters, VR also enables exploration of autonomic alterations, adding to an accurate assessment of social performance. Threat interpretations are associated with elevated cardiovascular responses in adolescents in ambiguous social situations [42], raising the question whether adolescents experiencing PLEs may show differing autonomic responses and more so when ambiguity increases. Research on autonomic arousal in individuals with and without psychosis is rare and inconsistent [43] and non-existing in youth populations experiencing PLEs. Exploring how ambiguity may affect social performance in virtual interactions can improve understanding and identification of at-risk individuals and inform potential interventions for this group. Despite previous VR-based assessment of components of social performance in individuals experiencing PLEs such as paranoid thoughts [27] and a recent study exploring paranoia in an adolescent population in VR [44], to date there is no VR paradigm integrating subjective, behavioural and physiological parameters to assess components of social performance, as well as the potential role of social stressors, in adolescents with PLEs.

## The present study

The present study is, to our knowledge, the first to explore the association between PLEs and cognitive, emotional, behavioural and physiological components of social performance (i.e., social anxiety, social avoidance, fear of negative evaluation, self-confidence, interpersonal distance, heart rate pulse and variability, and skin-conductance during social interactions) in a sample of adolescents aged 13–18 in VR. Since adolescents experiencing hallucinations and delusions may pose a distinct PLE subgroup in need of better understanding [14] and given the positive predictive value of these PLEs in screening instruments [12], two groups were defined based on survey responses: those reporting hallucinations or delusions, “highly-indicative PLEs” (HIP) and those reporting other or no PLEs (no-HIP). A novel paradigm in virtual reality, adapted to adolescents, allowed for a real-time assessment of both subjective and objective measures in an immersive, interactive, and highly controlled virtual world. We hypothesized that compared to the no-HIP group, the HIP group would report: (1) higher levels of social anxiety, social avoidance and fear of negative evaluation at baseline (i.e., in relation to their everyday life); (2) higher levels of social anxiety, social avoidance and fear of negative evaluation during virtual social interactions; (3) higher levels of anxiety, social avoidance, fear of negative evaluation, and lower self-confidence during more ambiguous virtual social interactions; (4) higher interpersonal distance, higher pulse rate, lower pulse rate variability and higher skin conductance during virtual social interactions; and (5) that these increases in behavioural and physiological parameters will be more pronounced in more ambiguous virtual social situations.

## Methods

### Study design and participants

This cross-sectional experimental between-group comparison study used a purposive sampling method. Participants were recruited from a longitudinal cohort study conducted by our research group (the Young Voices Research and Interventions Prospective Study, YVORI_Pro) [45] between March and June 2023. YVORI_Pro aims to assess German adolescents from North-Rhine Westphalia and comprises a total of 4 assessment points: baseline, 6-month follow-up, 12-month follow-up, and 24-month follow-up. The results of YVORI_Pro are expected to be published in the coming year. Participants included in the present study were recruited from those who completed the 12-month follow-up (*N* = 1064). Six of the seven schools involved in YVORI_Pro agreed to also take part in the VR experimental study. Three of these schools enabled recruitment and data collection at the school, whereas the other three schools referred consenting participants to come to the university department VR lab.

Invitation to participate in the present study was based on pre-defined stratification criteria following self-reported PLEs at the 12-month follow-up assessment of the YVORI_Pro study in November and December 2022. Participants were informed at time of assessment of YVORI_Pro that they may be invited to a subsequent study. Eligible participants met the following criteria: (1) aged 13–19, (2) capacity to provide informed consent; and (3) sufficient language skills in German to participate. An independent research team member, not involved in data collection, screened participants for eligibility. A random sample of 658 students was then selected and invited to participate. All participants were asked to sign the study consent form, and participants under 16 years of age required an additional written parental consent to participate. Participants were excluded if they were unable to engage in research procedures. The study was approved by the Ethics Committee of the Faculty of Psychology, Ruhr University Bochum (632R1) and was preregistered on OSF [46].

### Pre-VR measures

Prior to entering the virtual environment, participants completed electronic self-report questionnaires to obtain information about school, class, age, sex (male, female or non-binary) and first language.

The *Psychotic Experiences Inventory (PEI)* is a novel 11-item self-report questionnaire, aiming to assess psychotic experiences (i.e., perceptual and ideational disturbance, diminished emotional expression and avolition), and designed by Kelleher and colleagues [5] based on their research and clinical experience [12]. With permission from the original authors, the questionnaire was translated to German and back-translated for the longitudinal YVORI_Pro study by an external editor. The original authors of the questionnaire gave final approval on the translated version of the inventory. The total score demonstrated excellent internal consistency (Cronbach’s α =.91) and very good convergent validity with the Community Assessment of Psychic Experiences 15 (*r* =.69, *p* < 0.001). The questionnaire is currently being used in the European YouthGEMS study (https://youth-gems.eu/). Groups included in the presented study were defined based on endorsement of items in this instrument: participants indicating at least one of the “highly indicative” PLEs in the last 12 months were allocated to the “HIP” group: if they endorsed “*true”* to at least one of the items following items: 3:“*I often feel suspicious of other people*,* like they can’t be trusted*”, 7:”*I hear voices or sounds that other people can’t hear*” or 9: “*I sometimes see people or faces*,* even though no one’s really there*”. All other participants were allocated to the “no-HIP” group.

The German version of the *Liebowitz Social anxiety Scale for Children and Adolescents* (LSAS-CA-SR), originally a clinician-administered interview [22] is a 24-item scale used to measure daily performance-related anxiety and avoidance. It includes a total of 24 items rated on a five-point Likert scale and was translated and back-translated to German by an external editor. This questionnaire has shown high internal reliability (*α* = 0.90-0.97) [21]. One-way random effects intraclass correlation coefficient values for LSAS-CA scores range from 0.89 to 0.94. The scale shows good internal consistency and convergent and divergent validity [22]“.

The German version of the *Brief Fear of Negative Evaluation (BFNE) Scale* was also used to measure this component of social performance. This 12-item scale (rated on a five-point Likert scale) has been shown to be valid and reliable (Cronbach’s *α* = 0.90) [23, 47] and has previously been used in youth populations to measure fear of negative evaluation [48]. The shortened version correlates highly with the original scale (0.96) [22]. It’s interitem reliability is *α* = 0.90 and test-retest reliability *α* = 0.75.

### VR environment

**Fig. 1:**
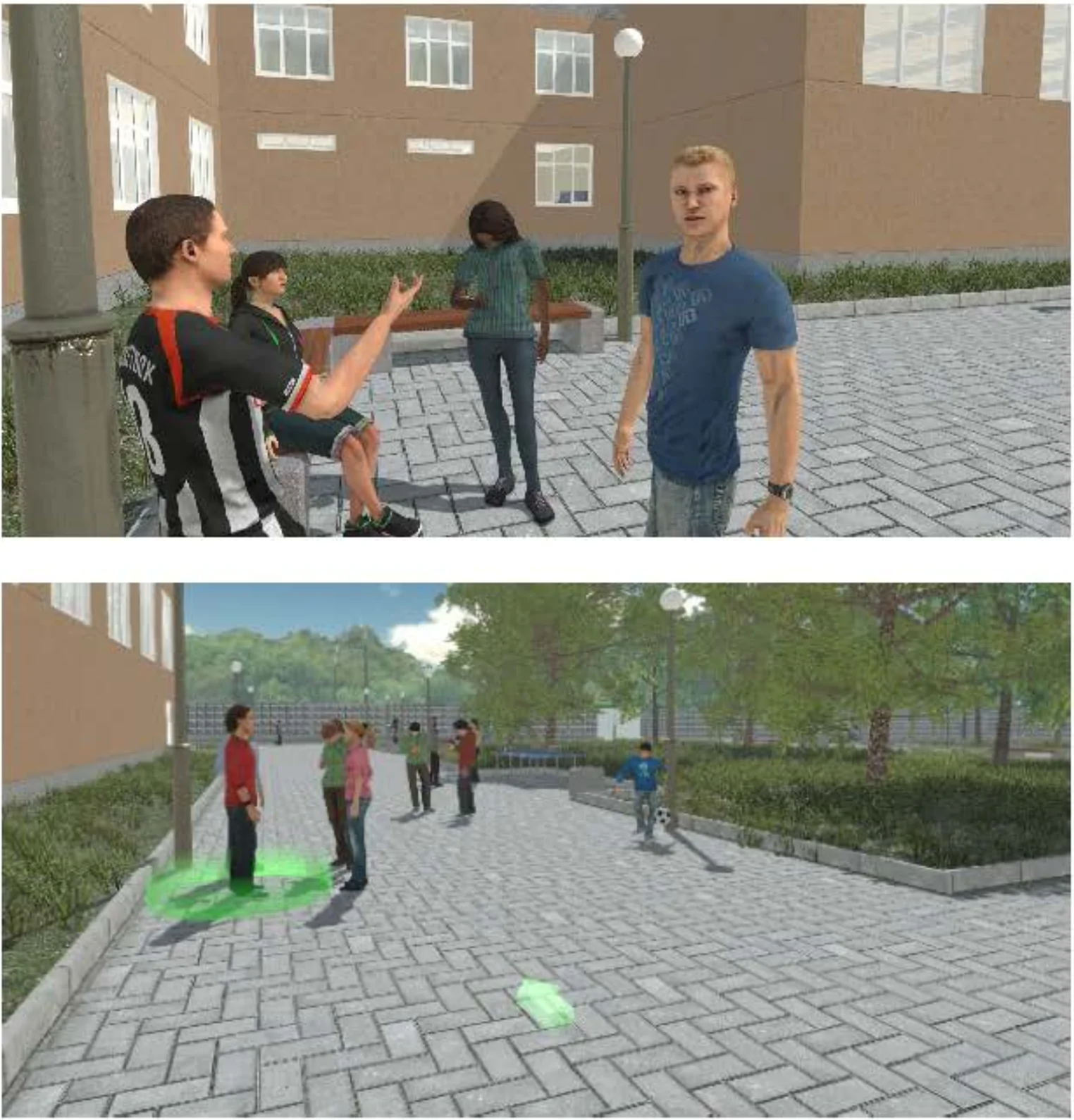
Screenshots of the virtual school yard environment

All participants experienced the same VR environment. The VR scenario depicted a school recreation area, featuring interpersonal interactions. Each scenario included adolescent avatars to align with participants’ expectations and enhance the sense of presence. The paradigm integrated advanced VR software (Unity) and a medical-grade wearable device, (Empatica EmbracePlus), to enable real-time acquisition of behavioural and physiological data. Participants experienced the virtual scenarios through a wireless head-mounted display (Pico 4), featuring a resolution of 2160 × 2160 pixels per eye and a 122.16° diagonal field of view, complemented by headphones. Navigation was controlled using a joystick. The school yard (see Fig. 1) had three different levels, varying in degree of social ambiguity. This was accomplished by manipulating two variables: crowd density and verbal comments made by avatars. Level 1 was nearly empty, with just 2 avatars in the far distance. Level 2 was crowded with 8 avatars and level 3 was densely crowded with 16 avatars. In level 1, lasting exactly 60 s, subjects were instructed to explore the environment. In level 2, lasting 170 s on average, subjects were instructed to approach 2 groups of 4 avatars. When participants approached avatars, some avatars looked their way briefly, others continued interacting. One of the avatars in level 2 made ambiguous comments to the subject such as “*shouldn’t you be studying for math’s?*” In level 3, lasting 220 s on average, subjects were instructed to approach 3 groups of 4 avatars. Two avatars also uttered ambiguous comments to the subject. Feedback from a lived experience advisory group and 17 pilot participants informed enhancements to the VR setting.

### VR-specific measures

Following each level of ambiguity in VR, four *Visual Analogue Scales (VAS)* on a five-point Likert scale, appeared. Adolescents were asked to rate their level of anxiety, “*how anxious do you feel?”*, fear of negative evaluation (FNE) “*how convinced are you of being negatively evaluated by the other people in the scene?*”, social avoidance “how much do you want to leave the current scene?”, and self-confidence “*how confident do you feel at this moment?”.* The answers ranged from “*not at all*” to “*very much*”.

A shortened 8-item version of the *State Social Paranoia Scale* (SSPS) [49] adapted for adolescents [33] was used to measure paranoid thoughts during the virtual interactions. Five items assess paranoid thoughts, and three items assess positive views of the avatars, which were reversed for scoring; items are rated on a five-point Likert scale. The originally 20-item questionnaire shows high validity and internal reliability (Cronbach’s *α* = 0.91) [49]. Participants completed the scale after experiencing all levels of social ambiguity in VR.

The 11-item *Social Comparison Scale (SCS)* [50] was used to measure perceived social rank. Items are rated on a ten-point scale. It has shown high internal reliability (Cronbach *α* = 0.88) [50]. The higher the score, the higher the perceived social rank in relation to the avatars in VR. It was translated to German and back-translated by an independent editor. Instructions were adapted to our VR scenario in that participants were asked to complete the sentence, “c*ompared to the people in the virtual reality scenario*,* I feel…”*, followed by each of the dimensions.

During VR exposure, *interpersonal distance (IPD)* was measured at a rate of 10 Hz in centimetres from the centre of the avatars’ heads to the front of the participant’s head. The software calculated the average amount of time spent within 1 m to avatars in percent in relation to the total time spent in the scenario. The radius criterion was chosen based on previous VR research on interpersonal distance [40].

The German version of the *Igroup Presence Questionnaire* (IPQ) [51] was used to measure spatial presence, involvement of the user, and experienced realness. This 14-item scale (rated on a seven-point Likert scale) has shown high internal reliability (Cronbach’s *α* = 0.85) [51]. Participants completed the questionnaire after removing the VR equipment.

### Procedure

**Fig. 2:**
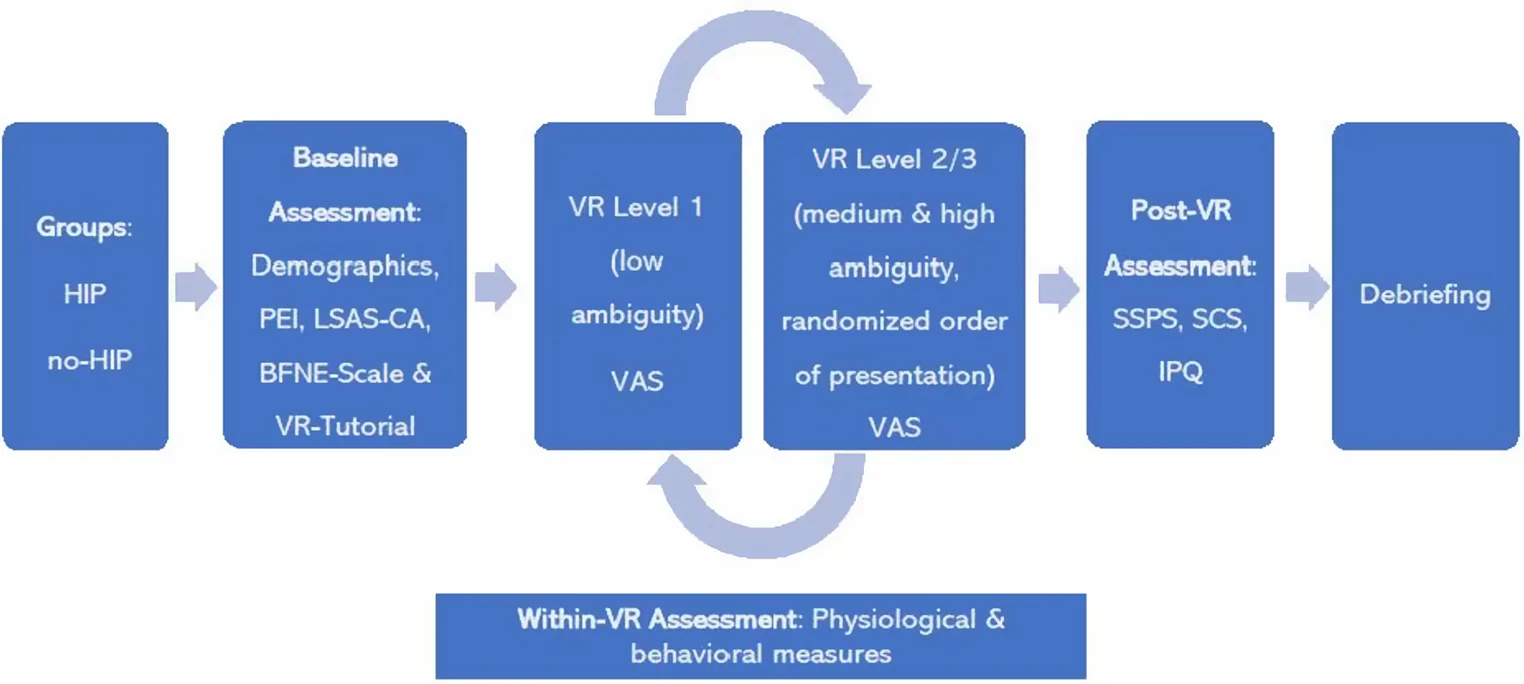
Virtual Reality Paradigm. HIP = highly-indicative PLEs, PEI = Psychotic Experiences Inventory, LSAS-CA = Liebowitz Social Anxiety Scale for Children and Adolescents, BFNE = Brief Fear of Negative Evaluation Scale, VAS = visual analogue scales, SSPS = Social State Paranoia Scale, SCS = Social Comparison Scale, IPQ = iGroup Presence Questionnaire

The researchers involved in the procedure and participants were blind to group allocation. All participants, and if appliable their parents, signed the study informed consent. After completing baseline questionnaires, participants entered a short VR tutorial before entering the virtual environment and moving through the three levels of social ambiguity. After each level, participants reported state level of anxiety, FNE, social avoidance and self-confidence using a virtual interface. All participants entered L1 before entering L2 or L3 to return to baseline (Fig. 2). L2 and L3 were presented in random order. Participants received a 10€ gift card for participating and, if applicable, 15€ reimbursement for travel costs.

### Data analysis

Analyses were conducted using R (version 4.3.2). To initially compare the individual measures per group and level, we either conducted independent t-tests or, if the data was not normally distributed (as checked for via Shapiro-Wilk-Tests), Mann-Whitney-U-Tests. Categorical variables were compared by conducting chi square tests. Missing data was omitted; this was only the case for physiological measurements.

Due to the sample size and only having two groups as well as its general robustness [52], ANOVA was determined the most appropriate test. Violations of sphericity were tested by conducting Mauchly’s tests and were accounted for by applying Greenhouse-Geisser (GG) corrections. Effect sizes were calculated using *η*^*2*^ [53]. A MANOVA test (with Bonferroni corrections) was performed to check for significant group differences in daily social performance. Levene’s tests were conducted to check for homogeneity of variance, and Box’s M test was conducted to check for homogeneity of covariance matrices for all dependent variables in the MANOVA. Repeated measures ANOVAs were conducted to examine the effects of group and level of ambiguity, as well as their interaction, on subjective, behavioural and physiological measures. All significant results were Bonferroni-corrected for pairwise comparisons. A Welch t-test was performed to explore group differences in sense of presence.

The VR application pre-processed raw data delivered by the EmbracePlus for the analysis of *pulse rate (PR)* and *pulse rate variability (PRV).* Pulse beat intervals (RR) were calculated using systolic peaks data delivered by the PPG sensor of the EmbracePlus, which measures data at 64 Hz. The root-mean-square of successive differences between RR intervals (RMSSD) was computed for every minute the participant spent in VR. Artefacts like missing heart beats were filtered by ignoring successive RR intervals during the calculation of the PRV if one of the RR intervals was more than 50% longer than the other one. PRV rates were interpolated to various timestamps for analysis. Lower PRV was associated with higher impairment.

To analyse *Skin Conductance (SC)*, range corrected scores were computed by using the minimum and maximum SC measured during the simulation. The participants’ SC at any other time period in the study could be delineated as a proportion of their individual maximum range of psychophysiological response via the formula (SC - SCmin) / (SCmax – SCmin) [54]. The values were thus normalized to values ranging between 0 and 1.

## Results

### Demographic characteristics

A total of 146 participants (35% identified as female, 65% as male and 0% as non-binary) were included in the final sample of the study (Table 1). Thus, the response rate was 22.1%. Most spoke German as first language (70% in HIP group and 81% in no-HIP group). In the HIP group, an average of 4.9 PLEs were reported, compared to an average of 1.3 in the no-HIP group. There were no significant group differences in terms of demographic characteristics.

**Table 1:** Descriptives and daily subjective social performance by group

	Total sample	HIP	No-HIP	
	*N* = 146	*N* = 89	*N* = 57	
PLEs	3.51 (2.71)	4.93 (2.38)	1.30 (1.37)	< 0.001
Age	15.66 (1.05)	15.70 (1.00)	15.61 (1.13)	0.654
Gender				0.102
Male	51 (34.93%)	26 (29.21%)	25 (43.86%)	
Female	95 (65.07%)	63 (70.79%)	32 (56.14%)	
Grade				0.667
9	62 (42.47%)	37 (41.57%)	25 (43.86%)	
10	47 (32.19%)	31 (34.83%)	16 (28.07%)	
11	37 (25.34%)	21 (23.60%)	16 (28.07%)	
First language				0.11
Bilingual	2 (1.37%)	2 (2.25%)	0 (0.00%)	
French	1 (0.68%)	1 (1.12%)	0 (0.00%)	
German	109 (74.66%)	63 (70.79%)	46 (80.70%)	
Kurdish	6 (4.11%)	3 (3.37%)	3 (5.26%)	
Other	14 (9.59%)	12 (13.48%)	2 (3.51%)	
Polish	2 (1.37%)	0 (0.00%)	2 (3.51%)	
Turkish	11 (7.53%)	8 (8.99%)	3 (5.26%)	
Ukrainian	1 (0.68%)	0 (0.00%)	1 (1.75%)	
Daily social performance				
LSAS-CA		48.52 (29.15)	28.35 (21.68)	< 0.001
BFNE		41.51 (14.71)	33.33 (11.48)	< 0.001

LSAS-CA: Liebowitz social anxiety scale for children and adolescents, BFNE: Brief fear of negative evaluation scale. Data are mean (SD) or n (%), unless otherwise specified. PLEs, age, gender, LSAS-CA and BFNE presented as *M*(*SD*)

### Self-reported components of daily social performance

There were significant group differences in cognitive and emotional components of daily social performance (*F*(1, 144) = 10.285, *p* <.0001, *η²* = 0.126). Post hoc tests indicated significant group differences in social anxiety LSAS-CA, *p* <.001 and FNE, *p* <.001, whereby the HIP group indicated higher levels of social anxiety and fear of negative evaluation than the no-HIP group (Table 1).

### Within-VR self-reported components of social performance

There were significant within-VR group differences in level of anxiety (*F*(1,144) = 13.63, *p* <.001, *η*²= 0.066), FNE (*F(*1,44) = 9.12, *p* =.019, *η²= 0.066)* and social avoidance (*F*(1,144) = 6.12, *p* =.014, *η*²= 0.031), whereby all values were higher in the HIP group There were no significant group differences in self-confidence. When level of ambiguity increased, anxiety (*F*(2,288) = 36.27, *p* <.001, *η²= 0.06)*, FNE (*F*(2,288) = 80.56, *p* <.001, *η²= 0.159)*, social avoidance (*F*(2,288) = 87.37, *p* <.001, *η²= 0.131)* and self-confidence (*F*(2,288) = 19.7, *p* <.001, *η²= 0.027)* changed significantly. No interaction effects were detected regarding group and level of ambiguity for any of the measures.

From L1 to L2, post hoc comparisons revealed significant increases in both groups in anxiety (HIP *p = <* 0.001, no-HIP *p = <* 0.001), FNE (HIP *p = <* 0.001, no-HIP *p* = < 0.001) and avoidance (HIP *p* = < 0.001, no-HIP *p* = < 0.001) and a decrease in self-confidence in both groups (HIP *p* =.005, no-HIP *p* =.01). Changes from L2 to L3 were not significant for any within-VR subjective measures. There was a significant group difference in perceived social rank, but not in paranoid attributions (Table 2).

**Table 2:** Within-and post-VR components of social performance

	HIP	No HIP	^a^
	*N* = 89	*N* = 57	
Within-VR			
Anxiety			
Level 1	1.67 (0.902)	1.18 (0.428)	< 0.001
Level 2	2.15 (1.15)	1.65 (0.79)	0.048
Level 3	2.25 (1.16)	1.70 (0.801)	0.006
FNE			
Level 1	2.18 (1.12)	1.49 (0.735)	< 0.001
Level 2	3.09 (1.18)	2.63 (1.2)	0.0620
Level 3	3.20 (1.36)	2.86 (1.25)	0.369
Avoidance			
Level 1	1.89 (1.03)	1.47 (0.758)	0.02
Level 2	2.75 (1.26)	2.30 (1.13)	0.111
Level 3	2.94 (1.33)	2.54 (1.27)	0.230
Confidence			
Level 1	3.38 (1.12)	3.56 (1.02)	0.915
Level 2	3.06 (1.23)	3.11 (1.1)	1.000
Level 3	2.99 (1.28)	3.07 (1.05)	1.000
IPD			
Level 1			
Level 2	0.32 (0.28)	0.38 (0.27)	0.370
Level 3	0.21 (0.20)	0.23 (0.20)	1.000
*p*	< 0.001	< 0.001	
PR			
Level 1	93.3 (13.9)	90.9 (16.2)	1.000
Level 2	95.1(15.7)	93.5 (15.4)	1.000
Level 3	97.3(12.8)	93.7(14.2)	0.792
PRV			
Level 1	93.3 (49.0)	96.1 (52.1)	1.000
Level 2	100 (46.4)	98.5 (47.3)	1.000
Level 3	98.2 (48.0)	101 (43.9)	1.000
SC			
Level 1	0.29 (0.35)	0.32 (0.32)	0.456
Level 2	0.59 (0.36)	0.66 (0.33)	0.767
Level 3	0.72 (0.30)	0.73 (F0.30)	1.000
Post-VR			
SCS	65.31 (16.82)	59.49 (16.79)	0.043
SSPS	12.27 (2.43)	11.65 (2.59)	0.155

IPD: interpersonal distance, *M(SD)* time spent within 1 m to Avatars in %, PR: pulse rate, PRV: pulse rate variability, SC: skin conductance, SCS: Social Comparison Scale, SSPS: State Social Paranoia Scale

^a^all p-values are Bonferroni-corrected except for SCS and SSPS

### IPD

Since participants were only instructed to approach avatars in L2 and L3, we only compared percentage of time spent within 1 m to the avatars for these levels. There were no significant group differences in IPD. There were significant differences in time spent within 1 m to the avatars depending on level of ambiguity, *F*(1, 144) = 63.13, *p* <.001, *η*² = 0.06. The interaction effect between group and level was not significant. Post hoc comparisons revealed that participants in both groups spent significantly less time within 1 m to the avatars in L3 (HIP *M* (*SD*) = 0.21 (0.20); no-HIP *M* (*SD*) = 0.23 (0.20) than in L2 (HIP *M* (*SD*) = 0.32 (0.28); no-HIP *M* (*SD*) = 0.38 (0.27), both *p* <.001.

### Physiological measures

Due to measurement errors during data collection, pulse rate data was missing for 12 participants from the HIP group and 3 participants from the no-HIP group. There were no significant group differences in pulse rate. Pulse rate varied significantly depending on ambiguity level, *F*(2, 280) = 12.561, *p* <.001, *η²* =0.0099 and results showed no significant interaction effect. Post hoc comparisons for pulse rate revealed significant increases from L2 to L3 for HIP (*p* <.001) and from L1-L2 in the no-HIP group (*p* =.041). There were no significant group differences in PRV. There were also no significant differences in PRV between levels of ambiguity. Skin conductance data was missing for 9 participants from the HIP group and 3 participants from the no-HIP group. There were no significant group differences in skin conductance. There were also no significant differences in skin conductance depending on ambiguity level.

### Sense of presence

There were no statistical differences between groups in total sense of presence (HIP *M* (*SD*) = 52.75 (10.54); no-HIP *M* (*SD*) = 52.77 (9.02)), t(132.3) = -0.012, *p* =.99.

## Discussion

To our knowledge, this is the first study to examine the association between adolescent PLEs and self-report, behavioural and physiological parameters of components of social performance using VR. We found that adolescents in the HIP group (those experiencing subclinical hallucinations and/or delusions) reported being more anxious, more avoidant and experiencing more fear of negative evaluation than adolescents experiencing other or no PLEs. As virtual social interactions grew more ambiguous, self-reported levels of anxiety, fear of negative evaluation and avoidance increased in both groups, with the HIP group consistently exhibiting higher levels of anxiety than the no-HIP group. There were no group differences regarding self-confidence, but it decreased in both groups when level of ambiguity in VR increased. Similarly, there were no group differences concerning proximity to avatars, albeit both groups exhibited increased distance to avatars with increased ambiguity. There were no significant group differences in physiological measures, and only pulse rate increased significantly in both groups when ambiguity increased.

The finding of higher levels of anxiety, avoidance and fear of negative evaluation in the HIP group conforms with previous research linking poorer social performance and PLEs [24–27], though this is the first time to demonstrate this relationship in adolescents. This finding also supports previous research in that the HIP group is a distinct PLE subgroup, which may have a higher risk of developing a psychotic disorder [14]. Nevertheless, although no-HIP participants appeared less anxious or fearful about the negative evaluation from others, it should not be neglected that other and negative PLEs (e.g. depressed mood) have also been shown to be relevant and practically meaningful in the prediction of at-risk states [55]. Also, since the HIP group indicated more PLEs on average, and the number of PLEs is an indicator of the risk of suffering from any psychiatric disorder [56], the HIP group may be at higher risk for psychopathology in general. This warrants further research on the prediction value of HIP for psychosis.

While prior research has established a causal relation between lower social rank and paranoia [28, 29], we found significantly higher perceived social rank reported by the HIP group and no significant group difference in paranoid attributions to the avatars. While poor functioning predicts risk of transition to psychosis in CHR individuals [17], it remains unclear whether the emotional and cognitive components of social performance reported by HIP individuals are comparable to those who have clinically identified as at CHR of psychosis, hence potentially at the beginning of an insidious deterioration of their social functioning. Considering that odd beliefs (i.e., delusional thinking) indicate higher psychosocial risks than anomalous perceptions (i.e., hallucinations) [9], distinct PLEs may indeed be linked to variations in poorer functioning. Distinguishing between these two experiences may improve risk prediction, yet exploring this was beyond the scope of this study and further research is needed.

In line with previous research [31], when subjective anxiety increased, IPD also increased in both groups. The more crowded the environment and the more ambiguous the avatars’ comments, the more distance adolescents kept to them, indicating that distress increased with increasing social ambiguity. While IPD has been used repeatedly in the past to differentiate individuals with and without psychosis in VR and has proven a valuable indicator of subjective state-dependent stress [40], IPD regulation in response to environmental social stressors has similarly been found unaltered in people with different levels of psychosis liability [38]. A complex virtual environment where many stimuli are present may cause attention to be divided, which might have reduced the tendency of adolescents with HIP to keep more distance.

In line with a previous study in which individuals with PLEs had comparable autonomic activity to healthy controls [43], we also did not find any group differences in physiological measures. This argues against an association of an alteration of arousal and vulnerability to psychosis, though previous findings are mixed [57, 58]. Our findings are limited by the fact that PRV is strongly influenced by breathing [59] and the participants in this study were standing and moving during assessment, which may present as a confounding variable. Previous research found that resting heart rate correlated with the presence of subthreshold psychotic symptoms and distress [60]; assessing this may therefore prove a more reliable and informative biomarker.

Following previous research showing that greater threat interpretations are associated with elevated cardiovascular responses in adolescents in ambiguous social situations [42], when social ambiguity increased, participants in both groups showed higher pulse rate. Contrarily, PRV and SC did not change significantly. The mixed effect of social ambiguity on physiological measures may be due to elevated SC acting as a warning signal when interpersonal distance does not suffice [61]. Since in our experimental paradigm, participants had the freedom to decide how long they wanted to stay close to the avatars, they may have sustained a regulated level of autonomic arousal. In this case, an experiment in which participants were forced to maintain proximity to others may have resulted in elevated arousal when social ambiguity increased but would have lacked freedom of personal choice. The increase in pulse rate when ambiguity increased, however, does indicate some increase in arousal [62]. Altogether, the data must be interpreted with caution since for 10.3% of the sample, the data was missing altogether.

The presented findings need to be regarded in the context of the study’s strengths and limitations. Using VR enabled observation of real-time interactions and controlled and safe manipulation of the environment. The triangulation of multiple data sources enabled a more accurate depiction of the participant’s experiences and responses. However, number of participants in each group differed slightly (i.e., more participants in the HIP consented to participate). Although participants were blinded to group allocation, those with positive PLEs may have been more interested in participation based on being more interested in the topic, which may limit generalizability of the study. Generalizability may be further limited since only two school forms were included which include mainly adolescents with a higher level of education (In Germany, different school forms cater to different educational paths and levels). Also, more females than males participated. Second, comparing individuals with only HIP vs. no-HIP meant we did not consider comparing also those adolescents reporting no PLEs though these may represent a distinct group. Third, due to its cross-sectional design, this study cannot clarify whether differences in functioning precede or follow the onset of PLEs, warranting longitudinal data to answer whether PLEs are also more persistent and recurring in those with HIP and impaired social performance Due to the outreach nature of the YVORI project, presence of psychotic experiences was self-reported and potential participants at a CHR of psychosis stage could not be clinically disregarded. Finally, using VR as assessment tool has its limitations. These include potential high costs, the possibility of inducing cybersickness in some cases, and variability in the degree of immersion depending on the specific type of environment used [30].

## Conclusion

The findings of this study provide evidence for the validity of this novel VR paradigm, which, to our knowledge, is the first to enable real-time, in situ assessment of cognitive, physiological, and behavioural components of social performance specifically in adolescents. The scenario elicited high presence and variability in outcome measures, demonstrating that self-confidence can be influenced in VR through social stressors like ambiguous comments and crowdedness. These results demonstrate that adolescents with PLEs can meaningfully engage with virtual social environments.

The findings also suggest that adolescents with specific PLEs, such as hallucinations and paranoid thoughts, show greater anxiety, avoidance, and fear of negative evaluation compared to other PLEs. While behavioural and physiological differences were not as prominent, emotional arousal was linked to IPD. Future research should explore the trajectories and risks associated with different PLE subtypes to better inform youth mental health interventions.

## Data Availability

The data that support the findings of this study are not openly available due to reasons of sensitivity and are available from the corresponding authors upon reasonable request. Data are located in controlled access data storage at the department for Clinical Psychology and Digital Psychotherapy, Bochum, Germany.
