# Correction: Exploring the association between adolescent psychotic-like experiences and components of social performance using a multi-level virtual reality paradigm

**DOI:** 10.1007/s00127-025-03009-9

**Published:** 2025-10-27

**Authors:** Grace Kiernan, Pauline Kohl, Ekincan Tas, Frederic Berg, Mario Wolf, Phuong-Mi Nguyen, Lucia Valmaggia, Mar Rus-Calafell

**Affiliations:** 1 Mental Health Research and Treatment Centre, Faculty of Psychology, Ruhr University Bochum, Bochum, Germany; 2 Department of Digital Engineering, Ruhr Uaniversity Bochum, Bochum, Germany; 3 Orygen, Centre for Youth Mental Health, The University of Melbourne, Melbourne, Australia; 4 Department of Psychiatry, KU Leuven, Leuven, Belgium; 5 Department of Psychology, Institute of Psychiatry, Psychology & Neuroscience, King’s College London, London, SE5 8AF UK; 6 German Center of Mental Health (DZPG), Partner Site Bochum/Marburg, Bochum, Germany

**Correction to: Social Psychiatry and Psychiatric Epidemiology (2025) 60:2023–2034**

10.1007/s00127-025-02871-x

In this article there is a small error under the demographics section in the results section, it is “65% identified as female, 35% as male”, but should have been “35% identified as female, 65% as male”.

The original article has been corrected.

